# **Investigation of Akt1 behavior on gold surface from molecular dynamics insight**

**DOI:** 10.1038/s41598-025-20366-2

**Published:** 2025-10-21

**Authors:** Farzane Abasi Joozdani, Mohammad Reza Amiran, Majid Taghdir

**Affiliations:** 1 Department of Biophysics, Faculty of Biological Science, Tarbiat Modares University, Tehran, 14115_111 Iran; 2 Behestan Innovation Factory, Tehran, Iran

**Keywords:** Gold nanoparticles, AKT protein, Electrostatic interaction, Molecular dynamics, Biochemistry, Biophysics, Cancer, Computational biology and bioinformatics, Drug discovery, Structural biology

## Abstract

In the past few years, gold nanoparticles (AuNPs) have shown great roles in biomedical areas. They can interact with proteins and change their structure and function. The serine/threonine kinase AKT plays a key role in cellular processes. Therefore, the AKT protein is known as a drug target for cancer treatment. In this study, we assessed the effect of gold nanoparticles on the AKT1 protein using molecular docking and molecular dynamics simulation. The results show that the AKT1 protein binds to citrate-coated gold surface predominantly through electrostatic and hydrophobic interactions. The RMSF calculations show that the AKT1 protein in the presence of gold nanoparticles exhibits less dynamic than the free state. The presence of gold nanoparticles causes the protein to have less compactness. The linker domain in the inactive conformation, and the regulatory domain and the glycine-rich loop in the active conformation of the AKT1 protein have higher dynamics than other regions. Furthermore, free energy landscape calculations show that AKT1 protein has a more conformational entropy in complex states in two active and inactive conformations. The results show that gold nanoparticles can affect the AKT1 protein and as a result, inhibit the phosphorylation flow in the protein signaling pathway in cancer cells.

## Introduction

 In recent years, nanotechnology has offered innovative strategies for detecting and controlling a wide range of biological processes occurring at the nanoscale, promising significant impacts on biology and medicine^1,2^. Among these, nanoparticles within the size range of biological molecules and structures have emerged as focal points of interest in biomedical research due to their potential applications^3,4^.

These nanoparticles can be engineered with advantageous properties to manipulate or detect biological structures and events effectively^5,6^. Gold nanoparticles are an important class of nanoparticles that have garnered considerable attention due to their unique physicochemical properties and versatile applications across various disciplines^6–8^.

On the other hand, proteins, as fundamental biomolecules, play vital roles in various biological processes. Understanding the interaction between proteins and gold nanoparticles presents significant implications in different fields from nanomedicine to environmental science^9–11^.

AKT protein as a protein kinase in the PI3K/Akt/mTOR pathway plays a key role in several cellular processes such as apoptosis, transcription, glucose metabolism, cell proliferation, and cell migration^12–14^. The AKT protein activation typically requires phosphorylation at two critical sites including threonine 308 (T308) and serine 473 (S473). Phosphorylation at these residues leads to conformational changes (The PH-in conformation to the PH-out conformation) that activate the kinase activity of AKT protein^15–17^.

The PH-in and PH-out conformations of the AKT1 protein refer to the structural changes in the pleckstrin homology (PH) domain of the AKT1 protein, which regulates its activity and subcellular localization. In the inactive state, the PH domain of AKT1 interacts with the kinase domain of the same protein, forming an auto-inhibitory structure. This is called the PH-in conformation. AKT1 is mostly cytoplasmic and inactive in this state because the PH domain masks the catalytic site, preventing AKT1 from being fully activated. The PH-in conformation keeps AKT1 closed, reducing its interaction with phosphatidylinositol (3,4,5)-trisphosphate (PIP3) at the plasma membrane. Upon activation by phosphoinositide-3 kinase (PI3K), PIP3 is generated in the plasma membrane. The PH domain of AKT1 has a high affinity for PIP3, and upon binding to PIP3, the PH domain moves away from the kinase domain. This movement exposes the kinase domain, resulting in the PH-out conformation. The PH-out conformation allows AKT1 to translocate to the plasma membrane, where it is phosphorylated at key residues (Thr308 and Ser473) by upstream kinases like PDK1 and mTORC2, leading to its full activation^18–20^.

The allosteric and ATP-competitive inhibitors are used in clinical treatments for inactive and active AKT conformations, respectively^21–23^. The AKT protein is comprised of three isoforms: AKT1 (PKBα), AKT2 (PKBβ), and AKT3 (PKBγ)^13,24^. The hyper-activated AKT1 protein is frequently observed in cancer cells undergoing migration^25,26^. Therefore, targeting AKT1 protein inhibition can be a therapeutic strategy for cancer treatment.

Various studies have indicated that gold nanoparticles exhibit anti-tumor and anti-proliferative properties against cancer cells^27,28^. This effect can be attributed to the generation of reactive oxygen species (ROS) by gold nanoparticles, leading to oxidative stress and subsequent cell death^29^.

In this study, we investigated the stability and structural changes of AKT1 protein in the presence of gold nanoparticles using computational methods. We prepared a gold nanoparticle sheet in which one surface was covered with citrate molecules. Then we evaluated the behavior of each of the two PH-in and PH-out conformations of AKT1 protein in the presence of gold nanoparticles.

## Results & discussion

**Fig. 1:**
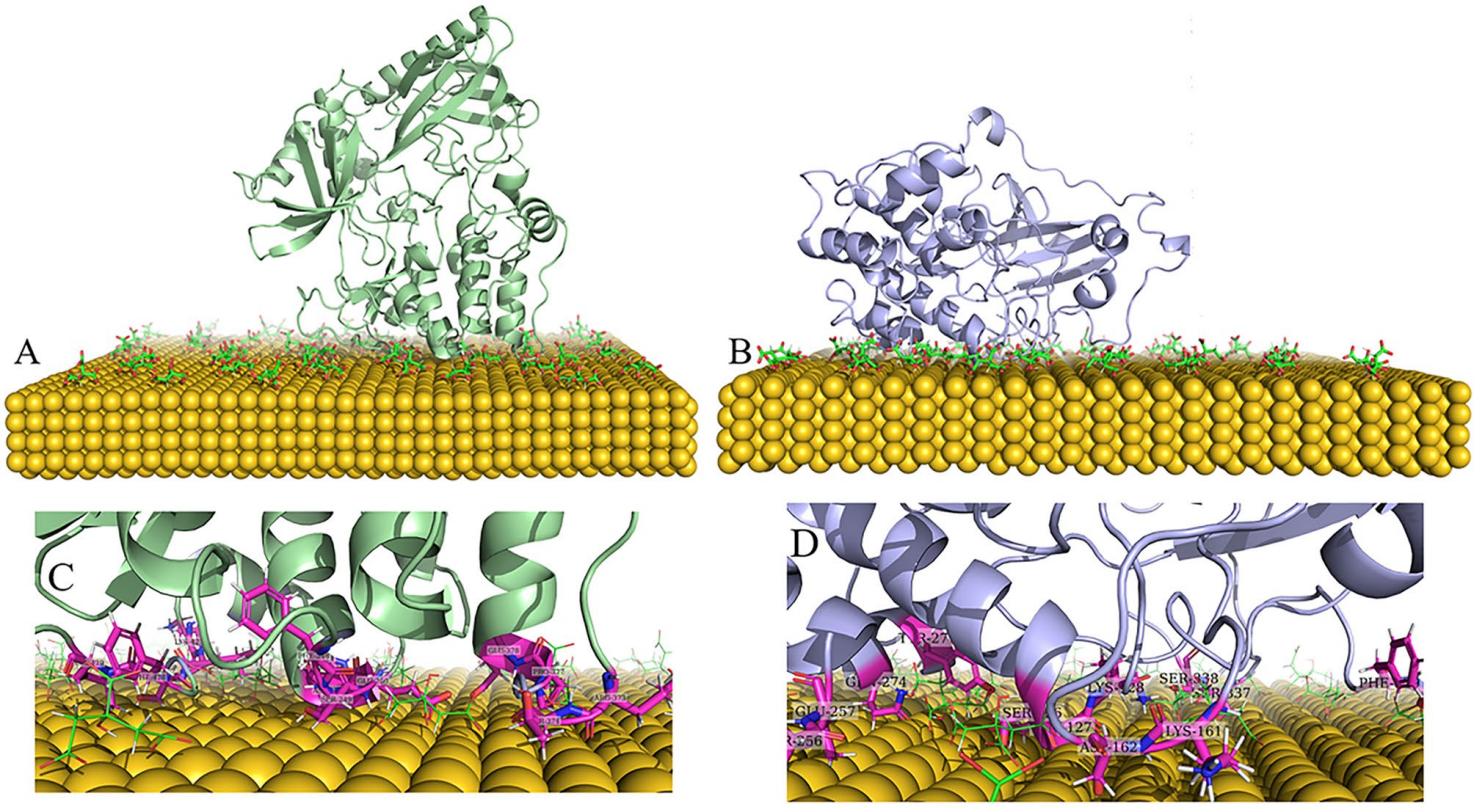
The 3D images of interactions between the AKT1 protein and the gold nanoparticle sheet were obtained from docking simulations (**A**, **B**, **C**, and **D**). The PH-in conformation (A, and C), and the PH-out conformation (B, and D).

The best binding modes between the AKT1 protein and a gold surface were successfully predicted through molecular docking using Patchdock. Figures. 1 A and 1B show the output of docking calculations.

Although the random arrangement of citrate on the gold surface reproduces the correct chemical composition and net negative charge, it does not represent the quasi-ordered and electrostatically stabilized arrangement observed in the experiment. In the real system, citrate ligands are usually relatively uniformly and flatly arranged, using all carboxylate groups for surface attachment; whereas random implantation can lead to unrealistic clustering, uncovered spots, and physically unacceptable orientations. STM and other scanning probe measurements have reported layers with detectable order, and molecular dynamics simulation also shows the formation of irregular but patterned “striped” or “island” domains rather than a completely random distribution. Since reaching equilibrium from a random arrangement is slow, short simulations may preserve these artifacts and affect reproducibility and local charge maps. Therefore, although random starting is computationally simple, a more structured initial arrangement or significantly increased equilibration time should be used to more realistically approximate citrate-coated surfaces^30–32^.

Citrate-coated gold surface usually have a negative charge due to the citrate molecule carboxylate groups. On the other hand, proteins can have negatively or positively charged residues on their surface. Therefore, electrostatic interactions between oppositely charged groups on the nanoparticle surface and the protein can play a key role in binding protein and gold surface. Furthermore, due to having hydrophobic areas on its surface, it can form hydrophobic bonds with the surface of citrate-coated gold nanoparticles. In addition, the presence of citrate molecules may cause the formation of hydrogen bonds between protein and citrate^33–35^.

In citrate-stabilized gold colloids—the dominant framework in biomedical experiments—the net negative surface charge not only prevents particle aggregation but also mediates protein adsorption and protein “corona” formation through similar electrostatic interactions. The same logic holds in our simulations: adsorbed citrate forms a negatively charged layer on gold that attracts positively charged spots on the protein. From a molecular perspective, the carboxylate groups of citrate can form salt bridges (carboxylate–ammonium pairs) with lysine residues of the protein. The consequence of this mechanism is that the protein binds primarily to the citrate layer, rather than to the bare gold surface, resulting in better preservation of native structure, more selective orientation, and more reversible adsorption. Therefore, citrate coating plays a pivotal role in determining the protein–surface interaction pattern in our system and closely reflects common laboratory conditions^36–41^.

The docking simulations revealed that charged residues on the kinase domain of AKT1 are pivotal in binding to the gold surface in both PH-in and PH-out conformations (Fig. 1C and D). This aligns with earlier studies showing that citrate-coated gold nanoparticles, with their negatively charged surfaces, preferentially interact with positively charged or polar residues on proteins^41–43^.

**Fig. 2:**
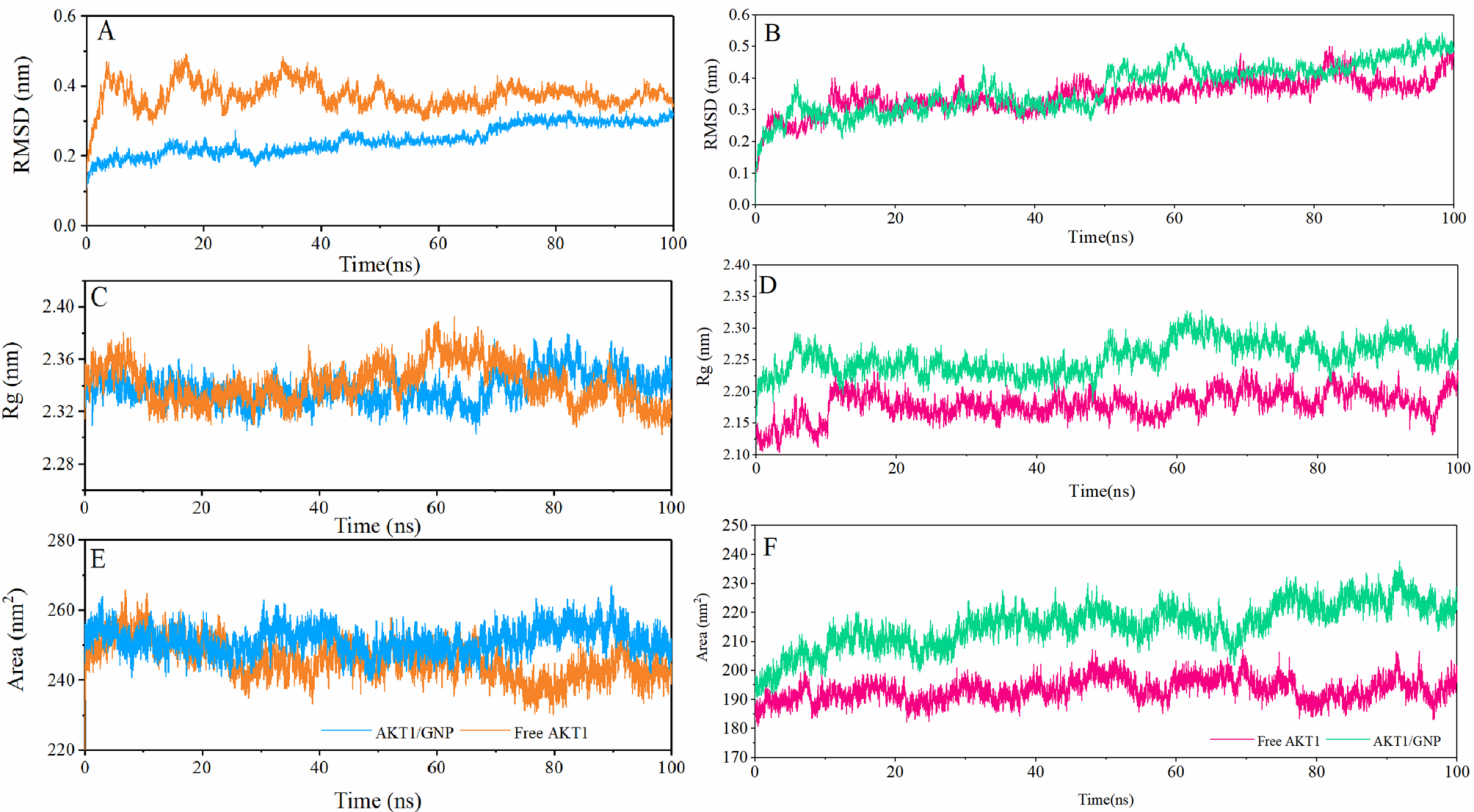
The RMSD of the AKT1 protein backbone atoms (**A** and **B**), the radius of gyration (**C** and **D**), and the Solvent-accessible surface area (**E** and **F**) of the AKT1 protein in the free state and interaction with gold nanoparticles during simulation time. The PH-in conformation (A, C, and E), and the PH-out conformation (B, D, and F).

The root mean square deviation (RMSD) of AKT1 protein backbone atoms in free state and complex is present in Figures. 2 A and 2B. According to Figure. 2, the AKT1 protein in PH-in conformations shows relatively higher stability in the presence of nanoparticles. This stability may result from strong and stable interactions between the protein and AuNPs, which limit large-scale structural changes. While in PH-out conformation, the AKT1 protein presented almost similar behavior in the first 50 ns of simulation, and in the continuation of the simulation the AKT1 protein in the free state had better stability than its complexed state (Figure. 2B).

The gold nanoparticles have a significant effect on AKT1 protein compactness. A comparison of the changes in the radius of gyration (Rg) of the AKT1 protein in each of the two conformations revealed that the proteins in the complexed states have less compactness than the free state. Changes in Rg in each of the systems were provided in Figures. 2 C and 2D. As shown in Figures. 2 C and 2D, the change in Rg in the free state of the PH-out AKT1 protein is significantly different from the complexed state.

The Solvent-accessible surface area (SASA) calculations confirm the change in the radius of gyration of AKT1 protein during simulation. The SASA calculations estimate the extent to which a protein is exposed to its surroundings. A lower SASA value indicates a more compact structure, whereas a higher SASA value indicates a more dispersed protein structure. Any change in SASA value signifies a shift in the protein’s structural conformation^44–46^. According to Fig. 2E F, the AKT1 protein in the free state has less SASA than the complex with gold surface. Therefore, gold nanoparticles cause the AKT1 protein to have a higher Rg and following that a higher SASA^28^.

This expanded state may reduce the efficiency of phosphorylation at Thr308 and Ser473 by increasing the conformational entropy and reducing the accessibility of critical phosphorylation sites. These structural changes suggest that gold nanoparticles disrupt the activation mechanism of AKT1 by destabilizing its active conformation and impairing key molecular interactions^28,47,48^.

**Fig. 3:**
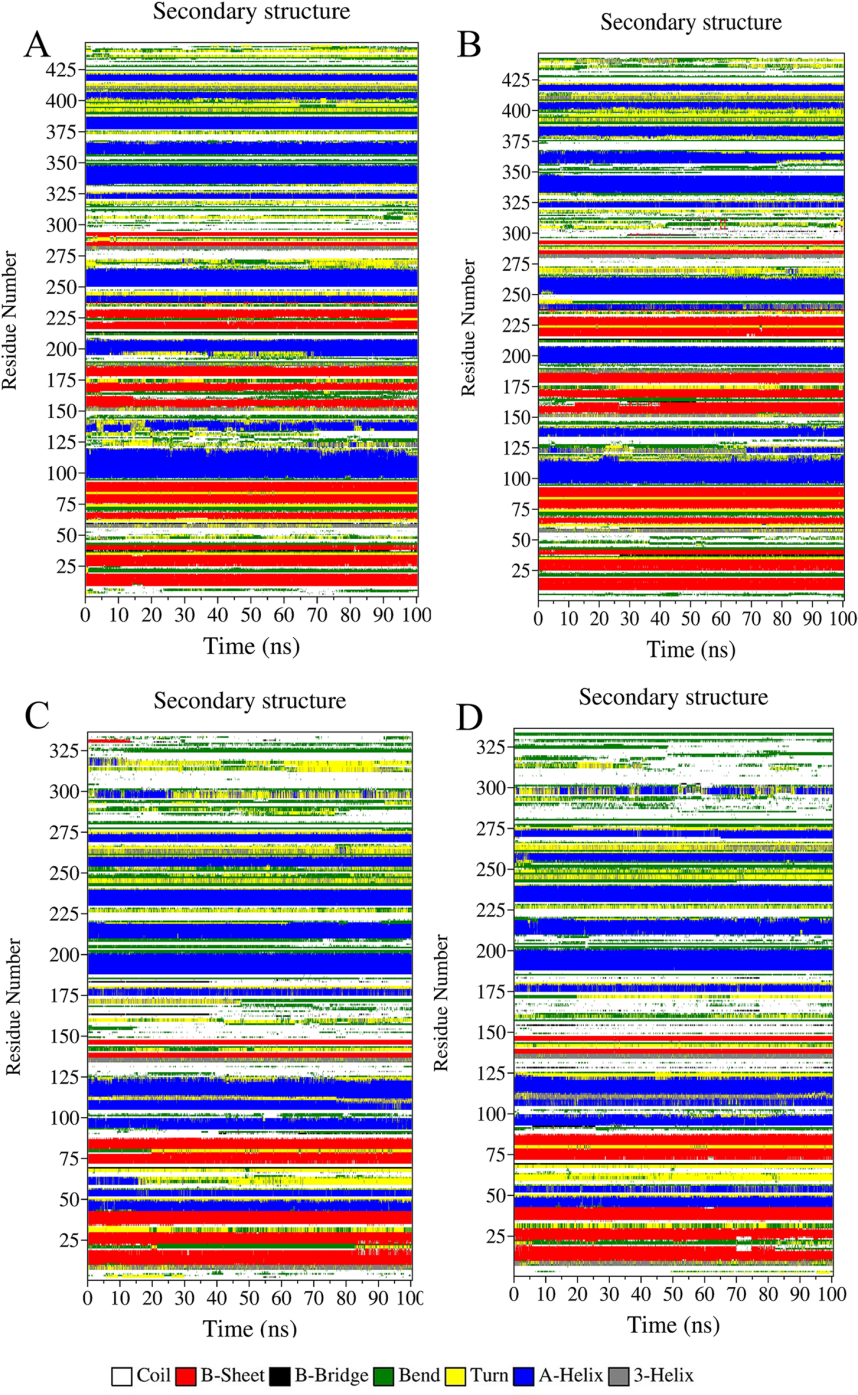
Evolution of the secondary structure elements of AKT1 protein in the free state (A and C) and interaction with gold nanoparticle sheet (B and D). The PH-in conformation (A and B), and the PH-out conformation (C and D).

On the other hand, the changes in the SASA impact the secondary structure of protein^49^. Figure. 3; Table 1 indicate changes in the secondary structure in each of the PH-in and PH-out conformations of the AKT1 protein which were measured by DSSP analysis^50^. In the presence of gold nanoparticles, the linker domain (108–150 residues) of AKT1 protein in PH-in conformation shows more changes than the protein-free state (Fig. 3A and B)^51^. In the PH-out conformation of the AKT1 protein, more secondary structure transitions especially in the C-terminal of the AKT1 protein happened during the simulation. These Changes are mostly related to the percentage Change of the coil structure, which has increased by 2% from the free state of AKT1 protein (Figures. 3 C, and 3D). The increase in coil structure in these regions suggests enhanced local flexibility, which could affect the protein’s ability to interact with upstream regulators or downstream effectors in cellular signaling pathways.

**Table 1:** Secondary structures percentage of the AKT1 protein in the free state and the presence of gold nanoparticles during 100 Ns molecular dynamics simulation.

Complex	Structure*	Coil	Β-Sheet	Β-Bridge	Bend	Turn	A-Helix	3-Helix
Free AKT1 (PH-in conformer)	0.56	0.25	0.19	0.01	0.15	0.12	0.25	0.03
AKT1/GNP	0.55	0.26	0.20	0.01	0.15	0.11	0.23	0.04
Free AKT1 (PH-out conformer)	0.51	0.33	0.13	0.01	0.14	0.12	0.25	0.03
AKT1/GNP	0.48	0.35	0.13	0.01	0.13	0.12	0.23	0.03

**Fig. 4:**
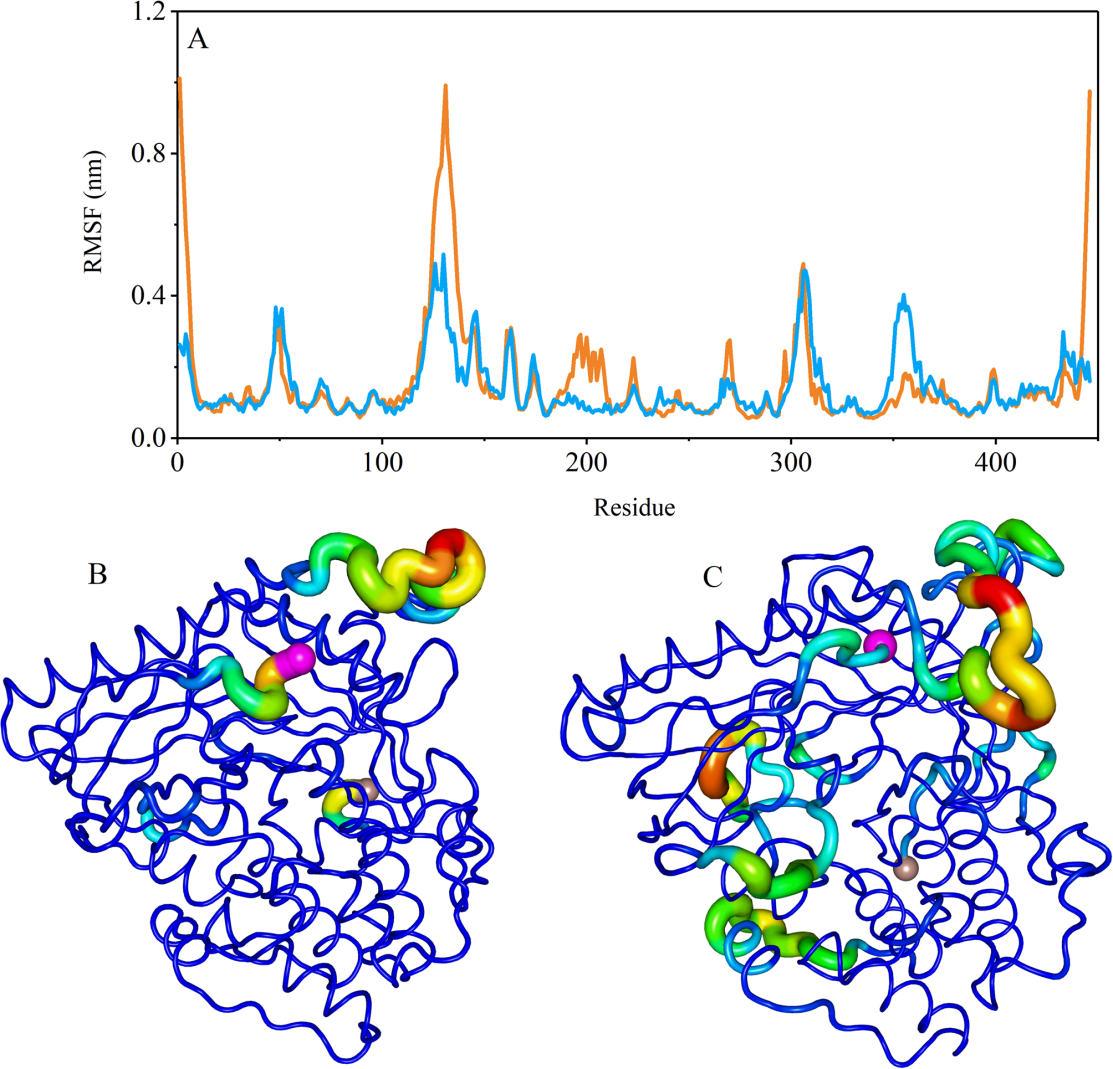
The RMSF values of Cα atoms of the PH-in conformation of AKT1 protein in the free state (orange color) and interaction with citrate-coated gold nanoparticle sheet (blue color) (**A**). The sausage representation for the AKT1 protein in the free state (**B**) and the presence of gold nanoparticles (**C**). The N-terminal and C-terminal of the PH-in conformation AKT1 protein marked with purple and brown spheres.

**Fig. 5:**
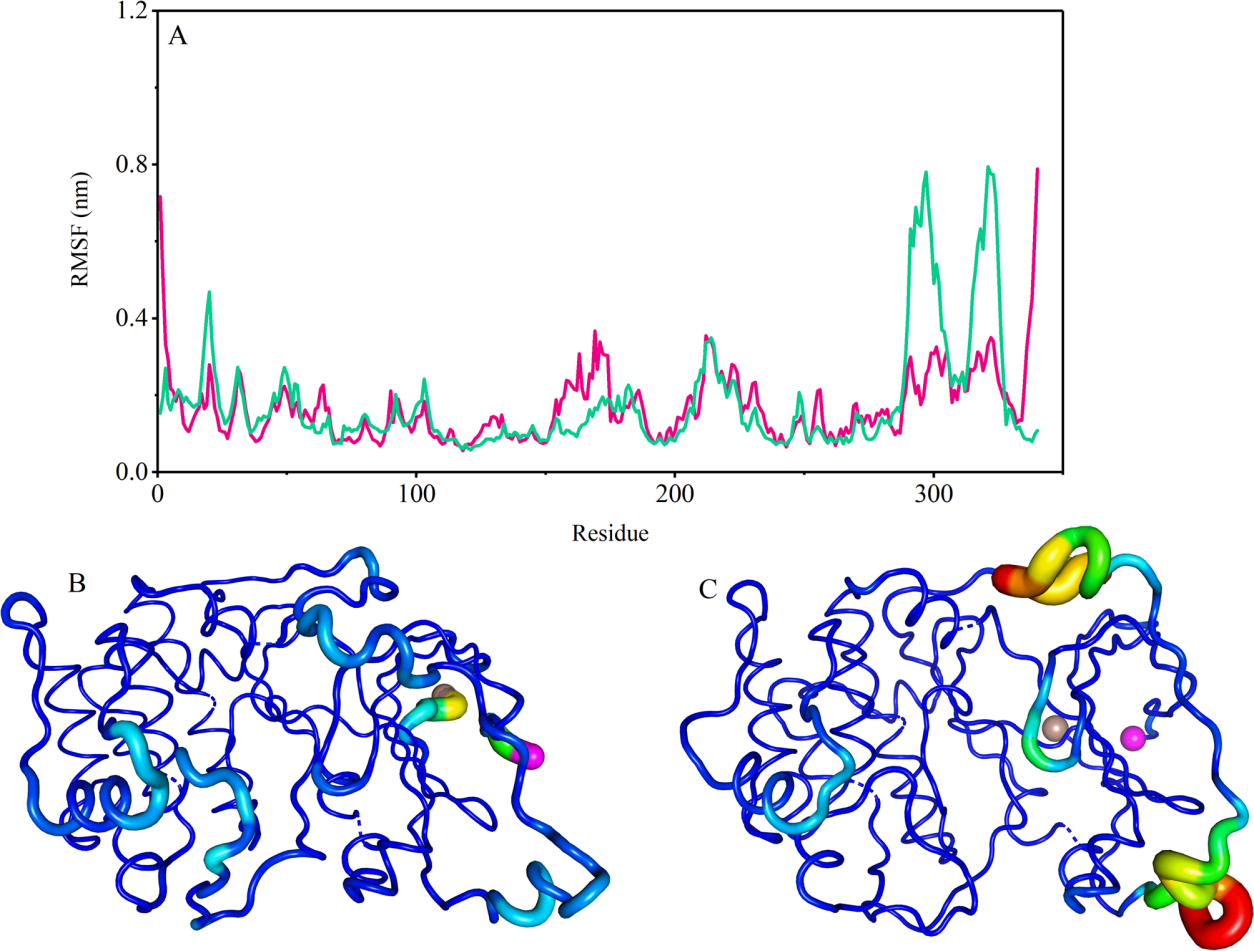
The RMSF values of Cα atoms of the PH-out conformation of AKT1 protein in the free state (red color) and interaction with citrate-coated gold nanoparticle sheet (green color) (**A**). The Sausage representation for the AKT1 protein in the free state (**B**) and the presence of gold nanoparticles (**C**). The N-terminal and C-terminal of the PH-out conformation AKT1 protein marked with purple and brown spheres.

**Fig. 6:**
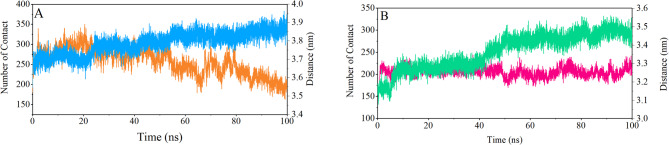
The distances between the centers of masses (COM) of the AKT1 protein and citrate-coated gold nanoparticles sheet (orange and red color), the number of contacts formed between the protein and gold nanoparticles (blue and green color) during 100 ns MD simulation. The PH-in conformation (**A**), and the PH-out conformation (**B**).

**Fig. 7:**
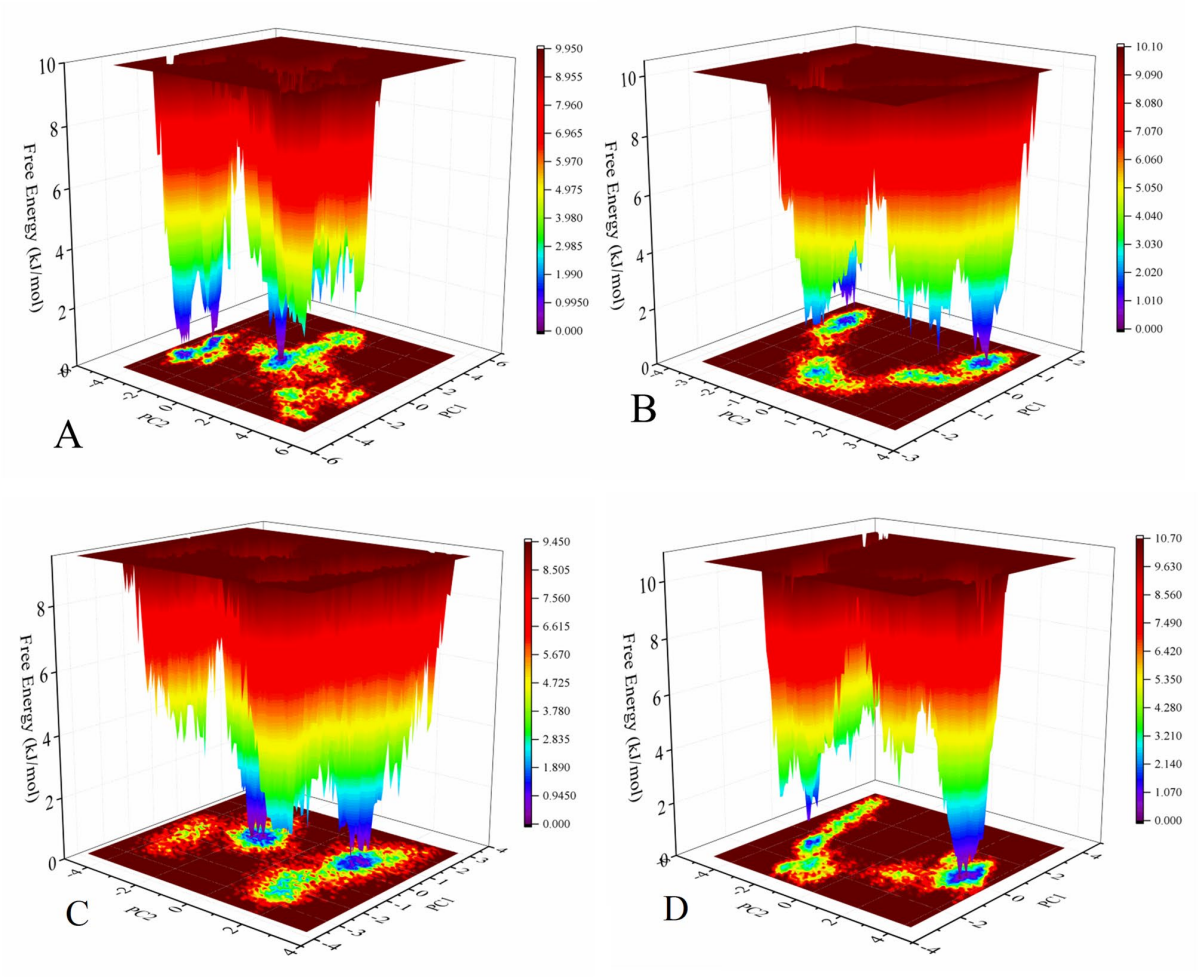
The 2D and 3D Free Energy Landscape (FEL) plots were acquired of the first two principal components during 100 ns MD simulations for the free state (**A, C**) and in the presence of gold nanoparticles (**B**, and **D**) of the AKT1 protein.

**Fig. 8:**
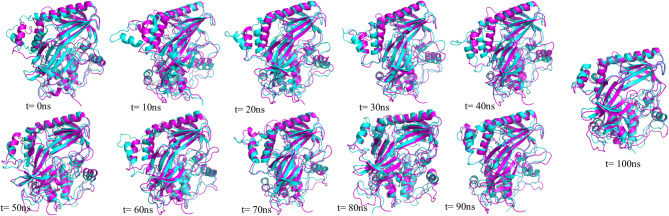
The superimposed structures of PH-in conformation of AKT1 protein in the free state and the presence of the gold nanoparticles with a 10 ns time interval. the free state (blue color) and the presence of the gold nanoparticles (purple color).

**Fig. 9:**
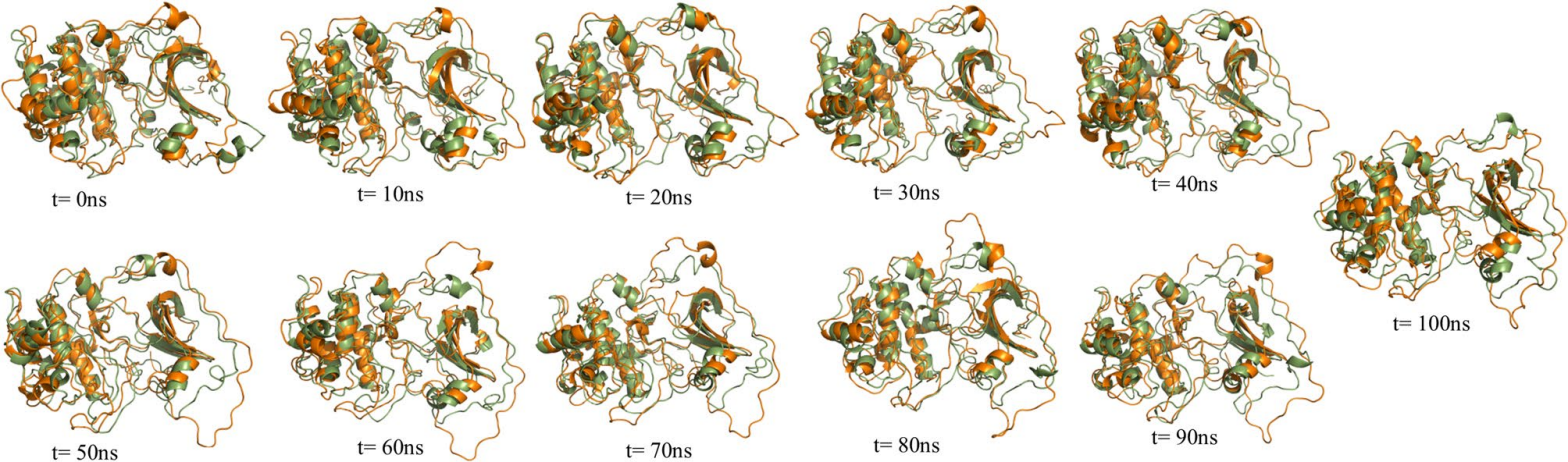
The superimposed structures of PH-out conformation of AKT1 protein in the free state and the presence of the gold nanoparticles with a 10 ns time interval. the free state (green color) and the presence of the gold nanoparticles (orange color).

The secondary-structure maps reveal that AKT1 maintains its overall protein-kinase catalytic domain architecture upon adsorption onto a gold surface, with only localised, orientation-dependent perturbations chiefly in flexible regulatory segments. The gold nanoparticle surface disturbs three separate, surface-exposed hot spots on AKT1 protein: (i) the phosphoinositide-binding edge of the PH domain (15–110 AA), (ii) the linker domain that connect the PH domain to the kinase region of protein (≈ 125–165 AA), and (iii) the activation-loop tip (308–325 AA) that including the regulatory Thr308 site. Because these parts are inherently mobile extensions of a rigid catalytic scaffold, their short transitions from helix/strand to bend/coil adjust membrane binding and phosphorylation kinetics without compromising the overall structure of the kinase itself. In the PI3K/Akt oncogenic cascade, AKT1 protein must first bind its PH domain to PIP3 protein in the inner layer of the plasma membrane and, after that, adopt an open catalytic conformation that exposes the activation loop threonine (Thr 308) and the hydrophobic motif serine (Ser 473) for phosphorylation. After modification of both sites, the kinase phosphorylates a large number of effectors that cause cell cycle progression and survival.

Figure 3 shows that a gold surface can switch this sequence at two points of occlusion without drastically altering the nature of the enzyme. When the PH domain surface is pressed onto the gold surface (Fig. 3B), residues 15–110 display alternating helix/bend motions, indicating the loosening of the first two beta strands from the edge of the PH domain, which is crucial for high-affinity lipid recognition. If such induced surface respiration were to happen in a cellular tissue, AKT1 would be less likely to bind to the membrane and slow access to the upstream kinase PDK1, therefore reducing pathway flux.

Also, a slight increase in flexibility at the tip of the activation loop (308–325 AA) and the PH-kinase interface (125–165 AA)—regions that must adopt precise geometries for orderly phosphorylation—would increase the energy penalty for holding Thr 308 and Ser 473 in a state suitable for phosphorylation. Fundamentally, the core of the protein-kinase catalytic domain, which is the site of the Lys 179-Asp 292 catalytic pair, remains intact, so gold nanoparticle binding is more likely to silence rather than catastrophically unfold AKT1 protein. Together, these structural cues align with emerging cellular data indicating that decreased AKT signaling leads to growth arrest and apoptosis^52,53^.

To indicate the effect of gold nanoparticles on the flexibility of each protein residue RMSF (the root mean square fluctuations) analysis was calculated. The calculations illustrated that the linker domain of PH-in conformation has higher flexibility than other regions. The linker domain exhibits greater structural shifts in the free state than the complexed state with gold surface. This suggests that the presence of gold nanoparticles restricts the flexibility of this region, potentially stabilizing its conformation. These regions were shown the higher B-factor values in the “sausage” plot (Figs. 4 and 5). In addition to the Linker domain, the residues 300–310 and 349–360, which interact more with gold surface, show dynamics and higher B-factor values.

In the PH-out conformation of the protein, the regulator domain residues show more flexibility than kinase domain residues^51,54^. The glycine-rich loop (aa 157–162) i.e. the region that ATP-competitive inhibitors interact with this region, in complex with gold surface showed higher dynamics and higher B-factor values than the free state of AKT1 protein^55^. Increased flexibility in the regulatory domain and glycine-rich loop in the PH-out conformation could disrupt interactions with upstream regulators (e.g., PDK1 and mTORC2) or downstream effectors, impairing AKT1’s role in the PI3K/Akt/mTOR pathway^56^. Moreover, this conformation C terminal region showed more Changes than the free state. The results of the RMSF measure are presented in Figure. 5. This analysis agrees with the above-mentioned analyses.

Distance between the centers of masses of gold surface and the centers of masses of AKT1 protein was evaluated by GROMACS distance command. Measurements show that the distance between the AKT1 protein in PH-in conformation and gold surface decreases with increased simulation time. (Figure. 6 A). Increasing the bond number between the AKT1 protein and gold surface confirms this distance decrease. In PH-out conformation, also, the number of bindings between the protein and gold nanoparticle sheet increases with increasing simulation time (Figure. 6B).

In MD simulation, the Free Energy Landscape (FEL) of the protein refers to the complex energy landscape that describes the different conformational states that the protein can adopt according to the corresponding free energy of that state. Various factors influence the FEL such as the sequence of the protein, its interactions with solvent molecules, and any ligands or cofactors bound to it^57,58^. To study the effect of the binding of the protein to gold surface on stabilizing or destabilizing the protein conformations, Changes in the free energy landscape were calculated. In this work, purple regions of the FEL analysis indicate lower energy levels compared to other regions. these regions represent the most stable conformations of the protein. As shown in Figure. 7, in the presence of gold nanoparticles, fewer energy basins were observed than the free state of the AKT1 protein. In these complexes, there is more conformational entropy than the free state of the AKT1 protein. Generally, the AKT protein in the presence of gold nanoparticles has a higher conformational mobility/flexibility, and lower structural stability.

Figures 8 and 9 show superimposed structures of PH-in and PH-out conformations of AKT1 protein in the free state and the presence of the gold nanoparticles with a 10 ns time interval. As seen in previous analyses, the linker region in the PH-in conformation, the glycine-rich loop in the PH-out conformation, and the regions that interact with the gold surface show more dynamic and structural changes during the simulation time.

Time-resolved superimpositions of AKT1 in the PH-in and PH-out conformations over timescales of 0–100 ns show that gold nanoparticles affect the interdomain dynamics of the protein without perturbing its secondary structure. In the PH-in conformation, the gold nanoparticles binding trajectory is significantly more compact and autoinhibited, such that the loop oscillations at the PH-kinase interface are reduced, peaking at around 60–90 ns. In the PH-out conformation, the divergence from the free state begins at around 40 ns and reaches its maximum at around 70–90 ns, where the gold nanoparticles-bound protein adopts a locally compact structure with finely oriented loops.

The increased dynamics in the linker domain (PH-in) and glycine-rich loop (PH-out) suggest enhanced localized flexibility, which may disrupt essential functional processes. The glycine-rich loop, critical for ATP binding and kinase activity, shows increased dynamics in the gold nanoparticles bound state, potentially impairing AKT1 catalytic function. Similarly, the structural destabilization of the regulatory and C-terminal regions in the PH-out conformation may hinder interactions with upstream regulators (PDK1, mTORC2) and downstream substrates, such as FOXO transcription factors and TSC2.

Collectively, these behaviors suggest that gold surface restrict the freedom of movement and orientation of the PH domain, making membrane recruitment and interaction with PIP3 unfavorable, and thus limiting access to PDK1 (Thr308) and mTORC2 (Ser473) for full AKT activation. The predicted functional consequence is that PI3K-AKT-mTOR signaling is attenuated (e.g., reduced phosphorylation of S6K and 4E-BP1), consistent with allosteric and reversible modulation, rather than denaturation. This interpretation is confirmed by the analyses mentioned above such as RMSF^52,59,60^.

Various studies and advancements have revealed the effect of gold nanoparticles on protein activity. These smart nanoparticles can have anti-tumor and anti-proliferative properties against cancer cells^28,43,61^. Gold nanoparticles interact with the biomolecules through non-bonded interactions, especially electrostatic interactions. They may also form a metal bond with protein if the protein has residues such as cysteine on the protein surface^33–35^. The reports show that gold nanoparticles induce apoptosis through inhibition of the AKT protein of the EGFR/PI3K/Akt signaling pathway in cancer cells^28^.

Docking simulations indicated that charged residues on the surface of the AKT1 protein have more contribution in binding to gold surface via electrostatic interactions^33^. The different analysis illustrates that the gold surface induce dynamics changes in the AKT1 protein in particular in the linker domain of PH-in conformation and the regulator domain and glycine-rich region of PH-out conformation. On the other hand, the structural and dynamic changes of the AKT protein in the presence of gold nanoparticles affect the solvent-accessible surface of the AKT1 protein and cause the SASA of the protein to increase. The structural and dynamic changes in the mentioned regions can prevent residue phosphorylation like T308 and S473. Therefore, it can disrupt the EGFR/PI3K/Akt signaling pathway in cancer cells and thus cause cell apoptosis.

Our simulations showed that gold nanoparticles induce significant changes in the structural flexibility of AKT1 in key regions, including the pH domain, glycine-rich loop, and activation loop. These structural changes may lead to impairments in AKT1 catalytic function and substrate accessibility. The pH domain, which is important for membrane binding, is subject to increased flexibility, which is likely to reduce the ability of AKT1 to bind to the membrane and interact with its upstream activator, PDK1. This change could disrupt the activation and phosphorylation processes of AKT1, ultimately affecting downstream signaling. Similarly, the glycine-rich loop, which is essential for ATP binding, becomes more flexible, which may negatively affect the ATP-binding pocket, leading to reduced coordination with ATP and reduced efficiency of the phosphorylation reaction.

In addition, the activation loop (residues 308–325) exhibits significant changes in flexibility, particularly in the PH-out orientation, which may alter the geometry of the active site and hinder substrate binding. RMSF and DSSP results also superimpose structural support for this hypothesis, indicating that these regions undergo significant displacements and become more dynamic upon interaction with gold nanoparticles. These structural changes in AKT1 are likely associated with reduced catalytic efficiency due to reduced substrate accessibility and impaired ATP binding. Therefore, our findings support the hypothesis that gold nanoparticle adsorption can affect kinase function through structural changes in critical regulatory regions^52,60,62^.

Different evidence suggests that our simulations provide a realistic representation of the gold–protein interaction and its potential impact on Akt signaling. Reports and cellular results suggest that contact of AKT1 signaling pathway proteins with gold surfaces can reduce Akt pathway output. In our work, the catalytic core of Akt remains intact, but the PH domain (along with the linker) and activation loop exhibit orientation-dependent flexibility that impairs membrane binding. The consequence of this impairment is a loss of PIP3-dependent uptake and reduced PDK1 accessibility; thus, Thr308 phosphorylation and downstream flux are reduced^52^.

This mechanistic picture is consistent with experimental observations: 3–5 nm gold nanoparticles reduced VEGF-induced Akt and eNOS phosphorylation and inhibited endothelial migration without apparent toxicity^63^. In a tumor cell models, treatment with gold nanoparticles attenuated growth factor-induced signaling and inhibited downstream activation of MAPK and PI3K–AKT cascades^64^. In breast cancer cell lines, AuNP-based nanoconjugates significantly reduced phosphorylated AKT1, simultaneously activated JNK, and ultimately induced apoptosis^62^. These reports suggest that AuNP can modulate AKT-dependent pathways and reduce their overall activity in cancer-causing contexts.

In a different study, Bin Wang and colleagues showed that dietary AuNP exposure in Drosophila increased the activity of the PI3K/Akt/mTOR axis and increased lipogenesis, a result that emphasizes that AuNP-kinase interactions can stimulate signaling rather than inhibit it, depending on biological conditions^48^.

At the molecular level, our simulation-based insights are consistent with previous computational reports of protein-gold binding. On citrate-coated gold surfaces, contact sites are predominantly provided by charged or polar residues—including lysine—and adsorption typically induces only minor perturbations in the protein architecture (like the areas mentioned above)^65,66^. According to RMSF profiles and superimposed structure, the protein retains its overall secondary structure and folding, and is more likely to undergo limited rearrangements or local unfolding. Furthermore, surface contact can rigidify some regions while allowing other regions to reorient—a pattern that has also been reported in simulations of albumin and transferrin on gold^67^..

Multiscale simulations on gold nanoparticles depict the adsorption pathway in three steps: collision, orientation, and extension of the side chains/rings; a process driven primarily by interfacial electrostatics and van der Waals forces. Key, this adsorption does not require global unfolding of the protein—a finding consistent with our DSSP maps and structural overlaps^68^.

The likely differences between the results are largely due to the severity and localization of these perturbations, which are themselves a function of nanoparticle curvature, ligand density/order, and the presence of a protein corona. These variables alter the interfacial electrostatic field and binding orientation. Therefore, our findings support the hypothesis that gold nanoparticle adsorption can affect kinase function through structural changes in critical regulatory regions.

## Computational details

### Gold nanoparticle sheet and AKT1 protein structures preparation

At first, a sheet of gold nanoparticles with an Au(110) surface orientation was generated by the CHARMM-GUI Nanomaterial Modeler module with dimensions 100 × 100 × 10 A°^69,70^.

When the nanoparticle size is much larger than the protein—for example, a gold nanoparticle with a diameter of ≳ 30–50 nm versus a protein footprint of ≈ 5 nm—the local curvature of the surface in the contact region is practically negligible, and from the protein’s perspective the surface appears “flat.” A natural consequence of this scaling is that an atomically flat gold facet can effectively represent the interface of a large spherical gold nanoparticle and reproduce key features of the protein–surface interaction.

Accordingly, in our molecular dynamics simulations, instead of modeling a perfect sphere, we used a well-oriented gold plate with a layer of adsorbed citrate to reflect the capping of the particle. This choice is also consistent with existing reports: previous studies have shown that flat gold surfaces reproduce ligand organization and protein binding patterns similar to those of curved nanoparticles with large radii. This common modeling strategy, while preserving essential protein–nanoparticle interactions, greatly reduces computational cost and allows us to simulate the AKT1–AuNP interface with high reliability on achievable timescales^71–75^.

Then one surface of the gold sheet was coated with 100 citrate molecules by using the GROMACS insert-molecules command. The obtained system was minimized for 5000 cycles using the steepest descent algorithm. The NVT equilibration step was performed for 1ns with time step 1 fs at a temperature of 300 K using V-rescale^76^. The citrate topology parameters were obtained by the SwissParam webserver (www.swissparam.ch)^77^. The INTERFACE force field was used on gold nanoparticles. This force field is compatible with the CHARMM36 force field^78^. The GRO file acquired from this simulation was used for its studying interaction with AKT1 protein.

The initial geometry of the AKT1 conformations for molecular docking simulations with gold nanoparticle sheets was taken from the 100 ns molecular dynamics simulation in our previous work^51^.

## Molecular docking simulation

The Patchdock web server^79^ was used to calculate and predict the best matching binding pose of the gold surface to two PH-in and PH-out conformations of AKT1 protein based on binding free energy. The last frame of 100 ns MD simulation of the AKT1 protein open and closed conformations in the free state and the last frame of simulation of citrate coated-gold nanoparticle was used for molecular docking calculations.

## Molecular dynamics simulation

All molecular dynamics (MD) simulations were performed with the GROMACS 2023 software package^80^. The parameterization of all molecules was done with the CHARMM36 force field^81^. MD simulation was separately carried out on each system with the same condition for 100ns. The structures obtained from the docking calculations were used to build the simulation box. The dimensions of the simulation box for all systems were adjusted to 10 × 10 × 12 nm. The TIP3P model was used to describe water molecules. Each of the systems was neutralized using Na^+^ and Cl^¯^ ions with a concentration of 0.05 M. Energy minimization was performed for each system by using the steepest descent algorithms to eliminate unfavorable overlap between neighboring atoms for 20,000 cycles. After that, two steps of equilibration (NVT, NPT) were performed by V-rescale^76^ and Parrinello-Rahman methods^82^ in 300 k and 1 atm during 2ns. During two equilibration steps position restraint of 1000 kJ mol^1^ nm^2^ was applied on the backbone atoms of AKT1 protein and gold nanoparticles and citrate molecules. The time step of 2 fs was set for the equilibration and production steps of MD simulations. The production step of MD simulations was performed in the NPT ensemble. The electrostatic interactions were calculated using the Particle Mesh Ewald method^83^ with a cut-off of 1.2 nm. Cut off of the van der Waals interactions was 1.2 nm with a switching function between 1.0 and 1.2 nm that was updated every 10 fs. The LINCS algorithm was implemented to constrain the bonds between atoms of the molecules^84^. The whole system trajectories were used to investigate the behavior of AKT1 protein in the presence of gold nanoparticles.

## Conclusion

In this work, we used computational methods to better understand the gold nanoparticles’ roles in the structure and dynamic Change of AKT 1 protein. According to docking calculation, non-bonded interactions especially electrostatic interactions have more role in binding the protein to surface. Our data of 100 ns molecular dynamics simulation show that in the presence of gold nanoparticles, the AKT1 protein has less compactness and a more solvent-accessible surface. The findings demonstrate how the dynamic and structural properties of the AKT1 protein are influenced by interactions, which primarily occur through electrostatic, hydrophobic, and hydrogen bonding forces. We hope these results can help using of gold nanoparticles for designing efficient inhibitors against cancer cells.

## Data Availability

The datasets generated during and/or analyzed during the current study are available from the corresponding author on reasonable request.
